# Attitudes Towards Clinical Research in Acute Illness—A Population‐Based Survey in Sweden

**DOI:** 10.1111/aas.70385

**Published:** 2026-10-27

**Authors:** Peter Bentzer, Niklas Nielsen, Moa Stenram, Fredrik Sjövall

**Affiliations:** ^1^ Department of Intensive and Perioperative Care Skane University Hospital Malmö Sweden; ^2^ Department of Clinical Sciences Malmö Lund University Lund Sweden; ^3^ Department of Clinical Sciences Lund Lund University Lund Sweden

**Keywords:** assent, incapacity, informed consent, intensive care medicine, legally authorized representative

## Abstract

**Background:**

To investigate the attitudes of the Swedish population towards participation in research and preferences regarding legally authorized representative when a participant is unable to give informed consent.

**Methods:**

A national survey conducted by a Swedish analysis and research company on a representative sample of the Swedish population on two occasions (2020 and 2023). The questions were based on the prerequisite that the respondents could not provide informed consent on their own. The questionnaire included two scenarios: one allowing time to consult a legal representative (non‐emergency) and one where research had to start immediately, before consulting a legal representative (emergency).

**Results:**

In total, 2086 respondents were included in the analysis. A majority were positive to participate in both non‐emergency (81% [1690/2086]) and emergency research (82% [1714/2086]) in situations where they would be unable to provide informed consent. Next of kin was preferred as a surrogate decision maker in 81% (1686/2086) of respondents and less than 1% (11/2086) preferred an unrelated legal representative such as an attorney.

**Conclusion:**

This study shows that in a representative sample of the Swedish population there is a positive attitude towards research in the acute setting and in critical illness, even in situations when a potential participant cannot receive information and provide informed consent and that a next of kin is the preferred legally authorized representative.

**Editorial Comment:**

This article reports results of a national survey from Sweden, where it shows that, among Swedish citizens, there appears to be broad acceptance of allowing family members to act as surrogate decision‐makers in informed consent decisions for study participation among acutely critically ill patients who cannot perform consent themselves. At this point in time, Swedish law does not allow this.

## 1 Introduction

Consent to participate in medical research should be free, voluntary and based on the participant being adequately informed, outlined in the World Medical Association (WMA) Declaration of Helsinki and the Oviedo Convention for the Protection of Human Rights and Dignity [1, 2]. The research participant should also be able to withdraw their consent at any given moment. In certain conditions, such as temporary or permanent mental disability, acute disease or similar, a person may not have the capacity to give informed consent. In critical illness, incapacity to consent is common due to the combined effects of the illness itself and treatments, such as sedation, which pose unique challenges for conducting research in the intensive care setting [3].

Recognizing the need for research also in patients with temporary or permanently reduced capacity to consent, the WMA Declaration of Helsinki states that research in those situations may be performed with additional safeguards considering the vulnerability of these persons. The prerequisites are that the potential participant may only be included if the physical or mental condition that prevents giving informed consent is a necessary characteristic of the research group AND the research is likely to benefit the participant OR if the research entails only minimal risk and minimal burden. In these situations, the researcher must seek informed consent from a legally authorized representative, who should consider the preferences and values expressed by the potential participant. If no such representative is available and the research cannot be delayed, the research may proceed without informed consent [1]. Informed consent, to remain in the study, must be obtained without undue delay from a legally authorized representative or from the participant, if they regain capacity to give consent. This framework for deferred consent was adopted by the EU Clinical Trials Regulation No. 536/2014 on clinical trials on medicinal products for human use that came into effect in the European Union (EU) in 2022.

When the European Clinical Trials Regulation was transformed into national legislation many EU countries, including all Scandinavian countries except Sweden, made provisions for a deferred consent process that allowed a relative to act as a legal representative, alone or in combination with an independent physician [4, 5]. In contrast, Swedish law requires the legal representative to be appointed by court order. This is a lengthy process involving several authorities, making it impractical in emergency research situations. It is currently unknown if the current legislation reflects the public opinion in Sweden. Observational studies in Finland and Denmark, continental Europe, United Kingdom, North America and Australia and New Zealand have suggested that patients and the general public support a deferred consent process but that acceptance rates vary (50%–96%) depending on the perceived risks with the research [6]. Less is known about stakeholder views on who should be their legally authorized representative in research situations, but the available evidence suggests that family members are the preferred representatives [7].

Based on the above, the presents study was designed to survey the public view in Sweden on consent to research both as a potential participant and a potential legal representative. For this purpose, we sent a questionnaire to a representative sample of the Swedish population at two time points 2 years apart.

## 2 Methods

### 2.1 Study Design

This was a cross‐sectional survey of a representative sample of the Swedish population performed by the poll institute Novus.

### 2.2 Questionnaires

The questionnaire was drafted by the authors and refined following feedback from Novus. Pilot testing was then performed on approximately 50 persons selected from the base panel (described below) to ensure understanding of the questions and further adjustments in the questionnaire if necessary. During the survey there were regular checks for comments on the panel support channel to address misconception. Response time of the survey was regularly checked for unreasonable quick response times and for participants who consistently responded ‘don't know’ which were then excluded from further analysis. All questions were mandatory to answer and thus there was no missing data. The questionnaire consisted of two sections, the first with clinical scenarios followed by questions and a second section collecting demographic information. This included age, sex, highest educational level (primary school, high school, college/university, did not finish school) and area of living (metropolitan area [cities with > 200,000 inhabitants with adjacent cities with at least 40% commuting to the metropolitan area], larger city [cities with > 40,000 inhabitants with adjacent cities with at least 40% commuting to the larger city], smaller cities and rural areas [cities with < 40,000 inhabitants]). The clinical scenarios described (1) research in a non‐emergency situation (i.e., time for the trial team to discuss with next of kin or legal representative) and (2) research in an emergency situation (i.e., no time for the trial team to discuss with next of kin or legal representative). The full questionnaire can be seen in the Supplementary Material. The answer options, for all questions except for Question 7 (Q7), were on a 4‐ or 5‐grade Likert scale with the gradings; *positive* to *negative* or *would like to participate* to *would not like to participate* [8]. *I don't know* was an option in all questions. Participants could only choose one answer in each question except for in Q7 where up to three options were allowed. The questionnaire was reviewed by fellow researchers, lay‐people and survey design experts at Novus, before distribution.

### 2.3 Data Collection Methods

Participants, aged between 18 and 79 years were selected from a base panel of 50,000 individuals kept by Novus, a Swedish analysis and research company. Both the base‐ and the participant panel of the current study were randomly selected, allowing for a representative sample of the Swedish population [9]. If a respondent had not answered the survey within 1 week, additional randomly selected invitations were sent until the minimal required number of responses was met. Initially, we planned to perform only one survey. However, the first was conducted during the Covid‐19 pandemic and to rule out that attitudes were affected by an ongoing pandemic, we post hoc decided to repeat the survey after the pandemic. The questionnaires were available for participants to answer on the web during approximately 3 weeks, from 30 October to 17 November 2020 and 20 September to 5 October 2023 and could only be answered once per participant.

### 2.4 Ethics

Opinion polls in Sweden are not subject to ethical review as the data collected is anonymous and not possible to trace back to an individual person. The questionnaire did not include sensitive personal data. Participation in the poll was voluntary.

### 2.5 Statistical Analysis

With 1000 individuals responding to each survey, the degree of confidence of finding the true population values were 95% (margin of error ± 2.5%) at a distribution of responses of 20/80 and 93.6% (margin of error ± 3.2%) at a distribution of responses of 50/50. Wilcoxon Signed‐Rank test was used to perform analysis between questions within the same questionnaire and between different years. For analysis of questions with answers on a Likert scale, ‘Don't know’ was not included in the comparison of positive versus negative responses. To avoid over‐representation, the question of preferred representative (Q7), all answers including a next of kin, were weighted and only counted as one. Univariable and multivariable ordinal regression analyses were performed to evaluate how demographic factors influenced the responses for having a next of kin giving consent (Q2) and willingness to participate in emergency research without prior consent (Q5). Results are presented as odds‐ratios with 95% confidence intervals (CIs). Statistical analyses were carried out using R 4.5.2 (R Core Team 2025) and IBM SPSS Statistics Data Editor version 26. *p* < 0.05 were considered statistically significant.

## 3 Results

### 3.1 Participants

The survey was distributed to a total of 4154 individuals (1843 in 2020 and 2311 in 2023) and 2086 individuals completed the survey (1069 in 2020 and 1017 in 2023), yielding a response rate of 50% (58% and 44% in the respective years). Responders were representative of the adult (18–79 years) Swedish population, including gender, age and education, referenced against data from, for example, Statistics Sweden and the Swedish Election Authority (Table 1).

**TABLE 1:** Demographics.

Variable	*N* = 2086
Sex	
Female	1020 (48.9%)
Male	1066 (51.1%)
Age (years)	
18–29	355 (17%)
30–49	707 (33.9%)
50–64	558 (26.7%)
65–79	466 (22.3%)
Educational level	
Primary school	119 (5.7%)
High school	760 (36.4%)
College/university	1204 (57.7%)
Did not finish school	3 (0.1%)

*Note:* Presented as *n* (%) or years.

### 3.2 Differences Between the Two Survey Time Points

The responses were similar between the two time points with the exception of Q1 and Q5, both relating to willingness to participate in research without prior consent, where more respondents were positive to participate in 2023 compared to 2020 (Q1: 83.3% [848/1017] in 2023 vs. 78.8% [842/1069] in 2020, *p* = 0.004; Q5: 82.6% [840/1017] in 2023 vs. 81.7% [874/1069] in 2020, *p* = 0.03). From here on, the results are presented as one combined cohort.

### 3.3 Attitudes Towards Non‐Emergency and Emergency Research When the Participant Is Unable to Give an Informed Consent

When asked about their own participation in research in a non‐emergency situation (i.e., time for the trial team to discuss with next of kin or legal representative, Q1), 81% (1690/2086) answered that they absolutely or likely would participate (‘Don't know’ 2.3% [47/2086]). In an emergency situation (i.e., no time for the trial team to discuss with next of kin or legal representative, Q5), 82% (1714/2086) were positive, answering they would absolutely or likely participate (‘Don't know’ 4.0% [83/2086]) (Figure 1). Thus, there was a small but significant numerical difference where more responders were positive to participate in the emergency—compared to the non‐emergency situations when deciding for oneself (Q1 vs. Q5, *p* < 0.001). An ordinal regression analysis suggested that female sex was associated with a more positive attitude to participate in emergency research (OR 1.19 [1.01–1.41] *p* = 0.042). Similarly, the attitude was increasingly more positive with age (yearly OR 1.01 [1.01–1.02], *p* < 0.001). Living in larger cities compared to metropolitan areas was associated with a more positive attitude to participating in emergency research (OR 1.43 [1.17–1.75], *p* = 0.002) (Table 2).

**FIGURE 1:**
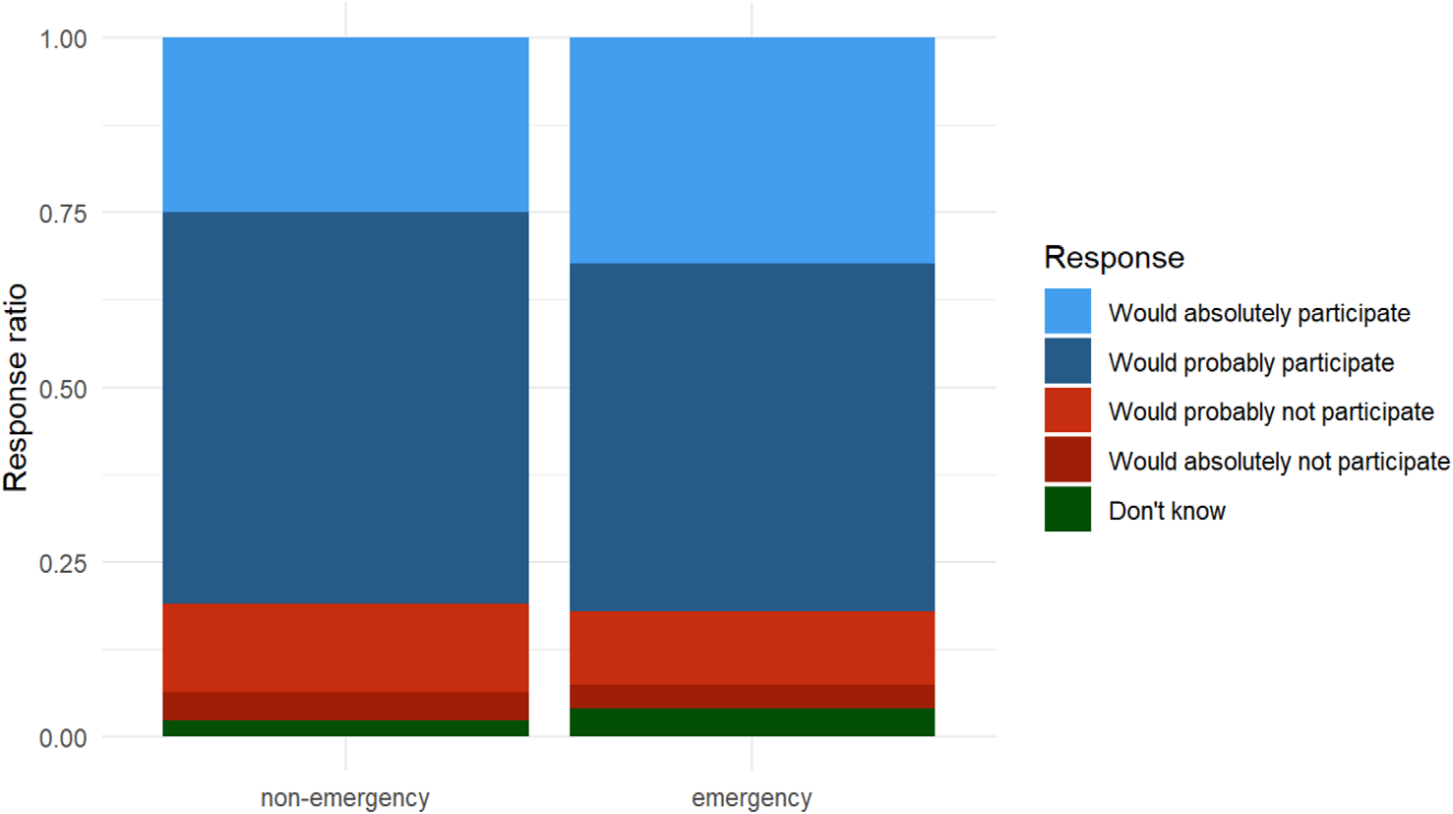
Response ratio on willingness to participate in non‐emergency and emergency research (Questions 1 and 5).

**TABLE 2:** Regression analysis of willingness to participate in emergency research.

Predictor	OR	95% CI	*p*
Age (years)	1.01	1.01–1.02	< 0.001
Sex			0.042
Male	—	—	
Female	1.19	1.01–1.41	
Educational level			0.5
Primary school	—	—	
High school	1.25	0.86–1.83	
College/University	1.27	0.87–1.83	
Did not finish school	3.89	0.41–84.3	
Region type			0.002
Metropolitan areas	—	—	
Cities	1.43	1.17–1.75	
Rural areas	1.23	0.99–1.51	

Abbreviations: CI = confidence interval, OR = odds ratio.

### 3.4 Attitudes Towards Legally Authorized Representative When the Participant Is Unable to Give Informed Consent

When asked if a next of kin could act as a legally authorized representative in a non‐emergency situation, when a research participant is incapable to consent (Q2), 88% (1828/2086) of respondents were positive 6% (121/2086) indecisive and 1.3% (28/2086) answered ‘Don't know’. In the emergency category (Q6), 85% (1764/2086) of respondents were positive, 8% (162/2086) indecisive and 1.8% (28/2086) answered ‘Don't know’ (Q2 vs. Q6, *p* < 0.001) (Figure 2).

**FIGURE 2:**
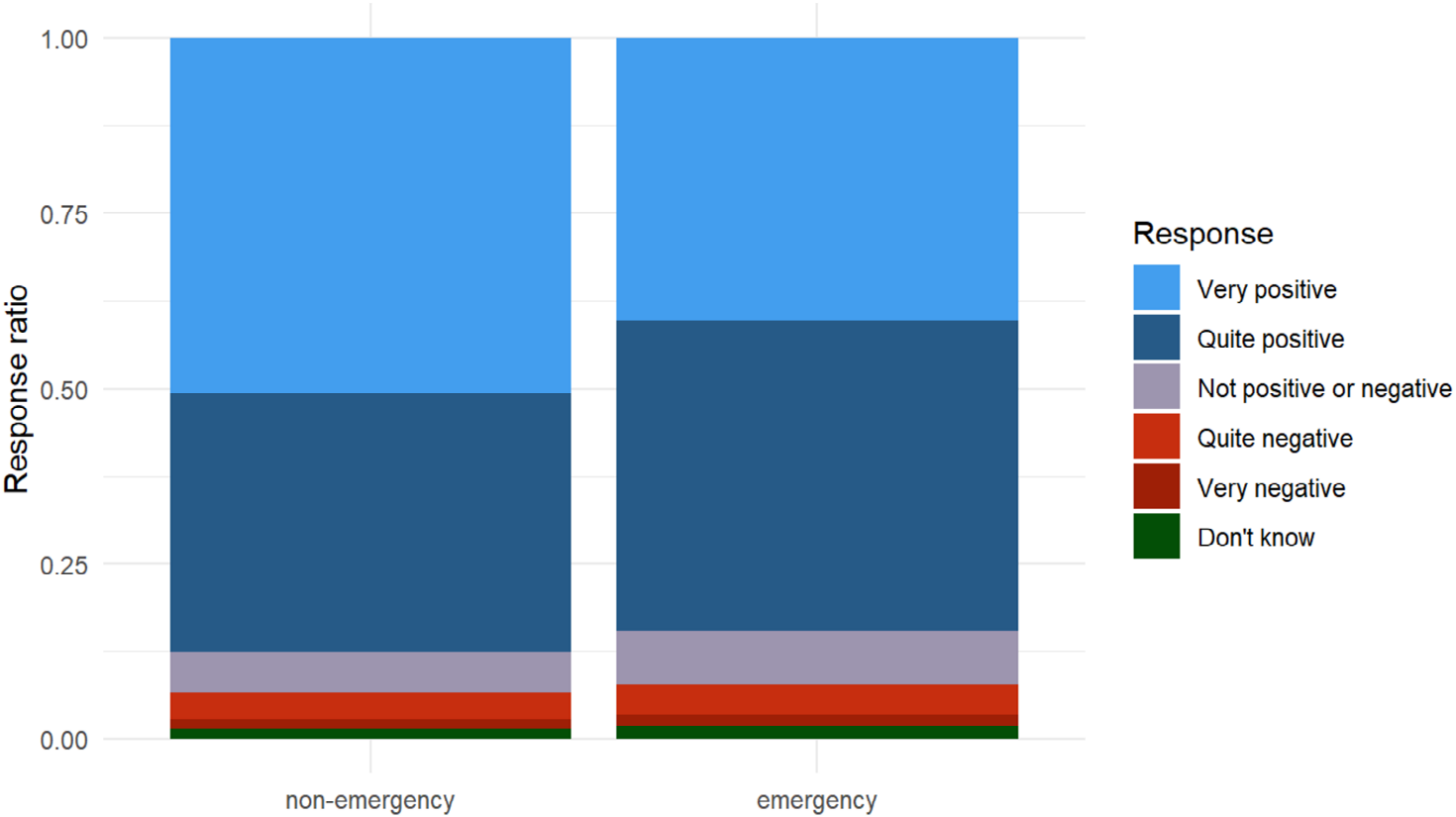
Response ratio on attitudes towards next of kin as provider of informed consent in non‐emergency and emergency research (Questions 2 and 6).

When responders were asked whom, they thought would be their most suitable representative in a medical research situation (Q7), 81% (1686/2086) answered with a next of kin (spouse/partner, child, parent or sibling). Responders were more positive towards a close relative than a more distant relative. Less than 1% (11/2086) of the participants answered with a legal representative such as an attorney as a preferred representative (Figure 3). A multiple regression analysis suggested that demographic differences including age, education or area of living did not influence the respondent's choice of person to represent their will (data not shown).

**FIGURE 3:**
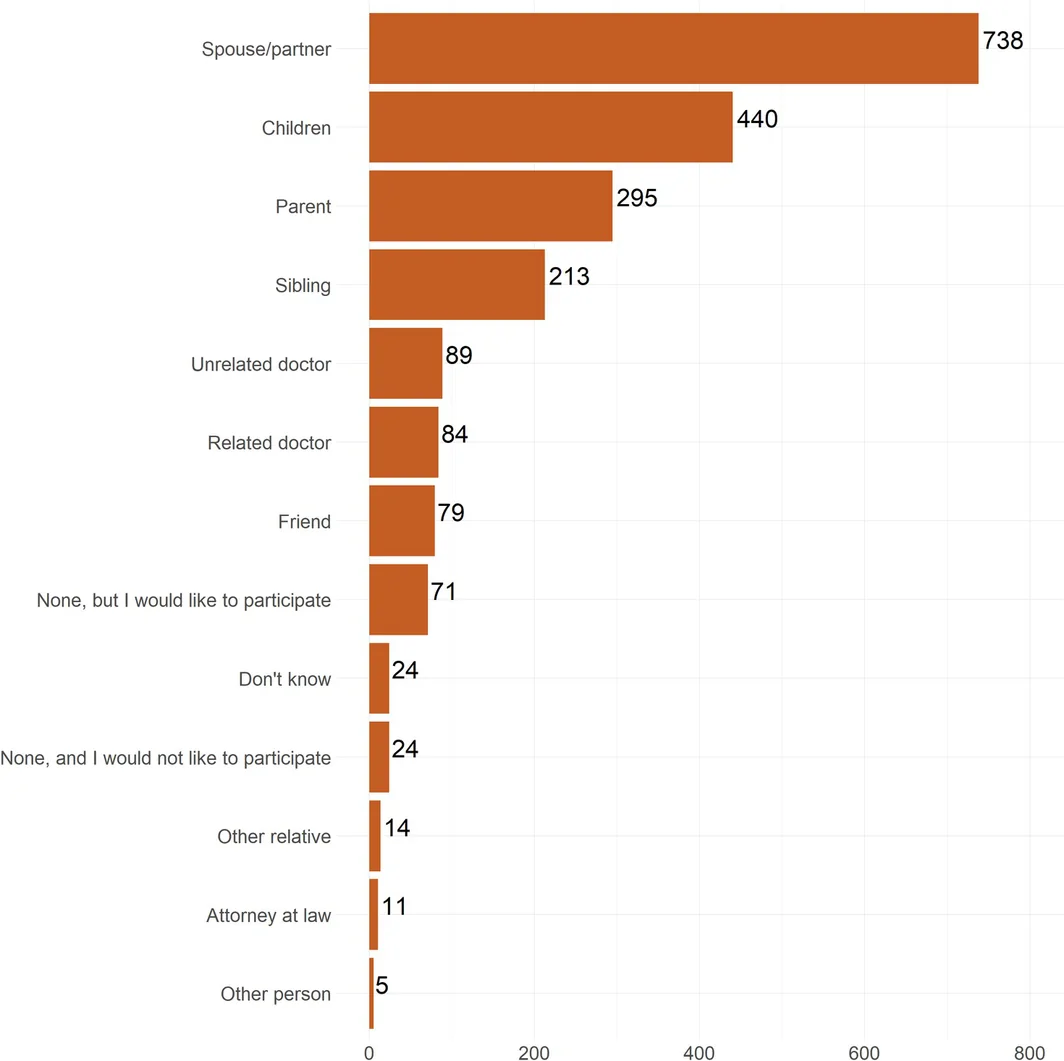
Number of responses to Question 7: Who, if anyone, do you think best represents your desire to participate in medical research in a situation where you yourself are unable to express your opinions?

## 4 Discussion

We performed two national surveys in the Swedish population 3 years apart on attitudes towards research in critically ill patients not able to give an informed consent. The results were similar between the two survey time‐points and show that respondents had a largely positive attitude towards participation in research in both non‐emergency and emergency situations. Most responders believed that a relative/next‐of‐kin would best mediate the will of the incapacitated individual. Very few preferred a legal counsel or other official representative.

Informed consent is a cornerstone of the Declaration of Helsinki and the Oviedo convention, both created to protect the autonomy of research subjects and not expose them to harm. Including participants unable to consent in research will always pose an ethical dilemma. However, not performing research in vulnerable individuals will lead to slower or absent increase of medical knowledge for these groups of individuals, hampering improvement in treatments and patient centred outcomes. Our finding that the majority of respondents in our Swedish survey were in favour of participation in non‐emergency and emergency research aligns with results in most studies performed in Finland and Denmark, continental Europe, United Kingdom, North America, Brazil, and Australia and New Zealand [7, 10, 11]. However, acceptance rates in previous research vary considerably (50%–90%) and in a small number of studies less than 50% of respondents find a deferred consent process acceptable [12, 13]. The differing acceptance rates likely reflect differences in how questions about deferred consent are presented, cultural context, as well as perceived risks with the proposed research [6]. Thus, even though our results add to a growing body of literature lending support for a deferred consent in emergency situations, given the provisions outlined in the declaration of Helsinki, the acceptability rate may be context dependent. It should also be noted that even though a majority of approximately 80% of respondents were positive in participating in emergency research, this still leaves one fifth not being positive. Thus, when formulating laws and regulations, as well as performing research, it is imperative that there are always means for potential participants or their legally authorized representative to opt out or withdraw from participation in research at any given time.

Many EU countries including all Scandinavian countries except Sweden have made provisions for a deferred consent process allowing a relative to act as a legally authorized representative when adapting national legislation to the European Clinical Trials Regulation. Most responders in our study believed that a relative/next‐of‐kin could best mediate the will of the incapacitated individual which aligns with data from several previous studies [14, 15, 16]. However, a large international study investigating public opinion (*n* ≈ 6000) reported that a physician was the preferred legally authorized representative suggesting that this result may be context dependent [17]. Very few (0.6%) of the respondents in the present study wanted to be represented by an externally appointed official such as a legal counsel and amongst family members, spouse or partner was a clear first choice for the majority followed by subsequently more distant relatives. When making a surrogate decision, the person appointed should be a representative of the research participant's will and try to set aside their own preferences as much as possible. We believe our results confirm the notion that the respondents believe that the more distant relation the surrogate has to the patient, the greater the risk of a patient‐surrogate discrepancy when making decisions. The ranking of relatives as preferred legally authorized representative in our study follows the same pattern as previously reported from the United States [15]. Even though our results clearly show that a next of kin is the preferred legally authorized representative in a Swedish setting it should be recognized that there are reports that participation in medical decision making by family members is associated with lack of comprehension, is stressful and many family members do not always wish to participate in those decisions. Also, some reports suggest that the accuracy of surrogate decision makers may be poor [18, 19, 20]. This suggests a need for thorough information and respectful communication with a potential surrogate decision maker [21].

Very few studies have analysed demographic factors associated with approval/disapproval of a deferred consent process. Our finding that female sex is associated with a more positive attitude towards approval of a deferred consent process contrasts with a study on participants and surrogate decision makers in a Canadian observational study [22]. Similarly, in a Brazilian study surveying opinions of ICU patients, male sex was borderline significantly associated with willingness to participate in a scientific study [20]. In that study, older patients (> 65 years) were less willing to participate in a scientific study, which contrasts with our finding that older persons were more likely to be positive to a deferred consent process. To our knowledge, analyses of attitudes in relation to living in a metropolitan area or non‐metropolitan area have not been studied before, and the finding with a more positive attitude for participation in research in the non‐metropolitan areas should be confirmed in future studies.

## 5 Strengths and Limitations

Strengths of our study include the large sample size representative of the Swedish population. To reduce bias, the panel was recruited using random population samples and no self‐recruitment was allowed. Moreover, the fact that we performed the survey both before and after the COVID‐19 pandemic with similar results supports their robustness.

Our study has several limitations worth commenting. First, the respondents were presented with hypothetical scenarios and questions, although based on real world scenarios, and it is not certain that the responders would have the same view when being exposed to an authentic situation, as it might be difficult to imagine oneself and a relative in such a situation. However, a high rate of consent to continue in the research projects among survivors in recent critical care randomized clinical trials, performed in Sweden, where a deferred consent approach was used suggests that the results are valid also in authentic situations [23, 24, 25]. The questions in our survey might also be biased towards certain answers even though the questions were formulated to be as neutral as possible and reviewed by several persons outside the study. We performed two surveys, 2 years apart and the fact that the first survey was distributed in the middle of the Covid‐19 pandemic when the public debate encompassed critical illness and clinical research may have affected the results. The second survey was distributed 3 years after the first and even though the medical system and life in general had since long returned to normal, we cannot rule out that there still have been affected by the pandemic. Since the results were very similar, we chose to present the data on the full cohort from both occasions. The participants in these surveys were randomly selected to represent the Swedish population. Instead of having a fixed number of participants to whom the survey was sent, we chose to use an iterating approach where more participants were added until the predefined number of responders was acquired resulting in a low margin of error which suggests that the results are representative of the attitudes in the general population. However, we cannot entirely exclude that those responding to a survey may differ from non‐responders thereby introducing bias. Another limitation is that we have not corrected analyses for multiplicity.

In conclusion, this study shows that in a representative sample of the Swedish population there is a positive attitude towards research in the acute setting and in critical illness, even in a situation when a potential research participant cannot receive information and provide an informed consent. The study indicates that the preferred legally authorized representative is a close relative.

## Author Contributions

P.B., N.N. and F.S. conceived the study and developed the questionnaires. M.S. performed initial analysis of the data and drafted the first version of the manuscript. All authors contributed to writing, editing and approving the final manuscript.

## Funding

The study was funded by The Swedish Research Council (F.S. and N.N.) and the Anna och Edwin Berger's Foundation (P.B.). The funding organizations have not been involved in the design, conduct, analyses or reporting of the trial nor will it have ownership of the data.

## Conflicts of Interest

The authors declare no conflicts of interest.

## Supporting information


**Data S1:** aas70385‐sup‐0001‐Supinfo.docx.

## Data Availability

The data that support the findings of this study are available from the corresponding author upon reasonable request.
