# Supplementary material for: Attitudes Towards Clinical Research in Acute Illness—A Population‐Based Survey in Sweden
Source: Acta Anaesthesiol Scand. 2026 Oct 27;71(1):e70385. doi: 10.1111/aas.70385 (PMC13713197; doi:10.1111/aas.70385)
Supplement: Supplementary file 1 [file AAS-71-0-s001.docx]

### Supplementary material

# **Attitudes towards clinical research in acute illness - a population-based survey in Sweden**

Peter Bentzer^1,2^*, Niklas Nielsen^3^*, Moa Jakobsson^3^, Fredrik Sjövall^1,2^

^1^ Department of Intensive and Perioperative Care, Skane University Hospital, Malmö, Sweden

^2^Department of Clinical Sciences Malmö, Lund university, Sweden

^3^ Department of Clinical Sciences Lund, Lund university, Sweden

* These authors contributed equally to this manuscript.

Corresponding author:	Fredrik Sjövall

[fredrik.sjovall@med.lu.se](mailto:fredrik.sjovall@med.lu.se)

### To English translated questionnaire

IT IS IMPORTANT THAT YOU READ ALL BACKGROUND INFORMATION BEFORE ANSWERING THIS SURVEY!

The questions concern topics in healthcare, treatment and research. Try to make a decision and answer the questions based on the knowledge and opinion you have right now; there are no right or wrong answers. If it is not possible to make a decision, you can always answer "do not know". We guarantee that any information you provide us with will be treated as strictly confidential. Protecting your privacy is just as important to us as it is to you.

VERY IMPORTANT!

Please read the upcoming background information carefully before answering the survey.

Medical research is being conducted to obtain better treatments and reduce the risk of side effects. It can be drugs that are to be studied or a new treatment method. All projects must be reviewed by an ethical review authority that assesses risks and benefits. If it is drugs that are evaluated, the projects are also reviewed by the Medical Products Agency.

The basic rule is that patients who are part of a research project are informed about the research as far as possible and are given the opportunity to consent to, or oppose, participation. In some cases, for example when a patient is unconscious or very critically ill, there is no opportunity to inform or ask the patient. Even in these situations, research is needed to develop and improve care.

In the Ethical Review Act, there is therefore an exception to information and consent where researchers in these situations can consult with a relative to try to form an opinion about the patient's attitude. The Medicines Act, on the other hand, is written in such a way that it is not possible to conduct research at all if the patient is acutely ill or unconscious. The treatments and drugs that we use for acutely ill people in Sweden are therefore largely based on research and knowledge from other countries.

In some of the subsequent questions, we ask you to imagine a situation where you have become so seriously ill that you cannot convey your will yourself (for example, in the event of a stroke, heart attack or serious accident) or where you are instead a relative of a patient who is seriously ill in a similar way.

**QUESTION 1**: In a situation where you are unconscious, how would you feel about participating in a medical research study, even though you cannot be informed about it or give your consent?

- Would absolutely want to participate
- Would likely want to participate
- Would likely not want to participate
- Would definitely not want to participate
- Don’t know

**QUESTION 2**: In a situation where you are unconscious, how would you feel about a next of kin trying to convey your will regarding whether you would like to participate in a medical research study or not? Your next of kin does not decide, but the researchers want to hear what they think you would like. In the legal text, this is described as a consultation.

- Very positive
- Rather positive
- Not positive nor negative
- Rather negative
- Very negative
- Don’t know

**QUESTION 3**: In a situation where you are unconscious, how would you feel about a professional, such as a lawyer or doctor, approving or rejecting your participation in medical research?

- Very positive
- Rather positive
- Not positive nor negative
- Rather negative
- Very negative
- Don’t know

**QUESTION 4**: *Introductory text of the question. In many countries in the EU, with the exception of Sweden, a next of kin can decide whether a patient, who is unable to convey his or her own will, can participate in a study or not.*

How would you feel about a next of kin approving whether or not you participate in a medical research study, if you are unable to communicate your will yourself? (Not just consultation, but your next of kin gives their consent or not for your participation).

- Very positive
- Rather positive
- Not positive nor negative
- Rather negative
- Very negative
- Don’t know

**QUESTION 5**: *Introductory text of the question. Imagine that you would have a cardiac arrest, be resuscitated but remain unconscious for a day before regaining consciousness. The ambulance has new equipment to improve the possibility of performing cardiopulmonary resuscitation. This equipment will be tested in a large study to see if it is safe and effective. In the past, smaller studies on humans and animal studies have shown positive results, but it has not been fully proven that the device is beneficial, perhaps it has no effect at all or it causes more side effects than benefit. That is why the research should be done. In order to evaluate the effect of the equipment, the ambulance staff must immediately decide whether you should take part in the study. There is no time to wait or ask a relative. The Swedish Ethical Review Authority has given its approval to the study.*

How would you feel about participating in such a study, even though you cannot be informed about it or give your consent because you are unconscious and there is also no time to consult with a next of kin?

- Would absolutely want to participate
- Would likely want to participate
- Would likely not want to participate
- Would definitely not want to participate
- Don’t know

**QUESTION 6**: What is your position on a next of kin trying to convey your will after inclusion whether you would like to continue participating in the medical research study or not? Your family member does not decide, but the researchers want to hear what your family member thinks you would like (retrospective consultation).

- Very positive
- Rather positive
- Not positive nor negative
- Rather negative
- Very negative
- Don’t know

**QUESTION 7**: Who, if anyone, would you think best represents your desire to participate in medical research in a situation where you yourself are unable to express your opinions?

Enter a maximum of three answers.

- Husband / Spouse / Partner
- Children
- Parents
- Siblings
- Other family member
- Friend
- A doctor I know (eg family physician)
- Independent doctor
- Legal counsel
- Other person than mentioned above
- There is no one to communicate my wishes, but I am open to participating in research
- There is no one to communicate my wishes, and I am not willing to participate in research

**QUESTION 8**: In a situation where a family member is not conscious, how do you feel about the fact that you, as next of kin, are trying to convey your relative's willingness to participate in medical research? You do not make decisions for family member, but the researchers want to hear what you think he or she would like (consultation).

- Very positive
- Rather positive
- Not positive nor negative
- Rather negative
- Very negative
- Don’t know

**QUESTION 9**: In a situation where a family member is not conscious, how would you feel about the fact that you, as next of kin, approve whether or not your family member can participate in medical research? (Not just consultation, but you give your consent for your family member to participate or not).

- Very positive
- Rather positive
- Not positive nor negative
- Rather negative
- Very negative
- Don’t know

**QUESTION 10:** *Introductory text of the question. Today, you can prepare a so-called power of attorney for the future, which gives next of kins the opportunity to take care of personal or financial matters if one party becomes unable to make decisions themselves, such as in the event of acute illness. However, it does not apply to medical issues.*

What is your opinion on the possibility of preparing a power of attorney where you can document in advance that a next of kin may decide on medical research-related issues if you are unable to decide yourself?

- Very positive
- Rather positive
- Not positive nor negative
- Rather negative
- Very negative
- Don’t know

**QUESTION 11**: Due to current legislation, certain medical research cannot be carried out on acutely ill patients in Sweden. What is your opinion on the fact that we use results that have emerged in other countries where the legislation allows such research?

- Very positive
- Rather positive
- Not positive nor negative
- Rather negative
- Very negative
- Don’t know

**QUESTION 12:** *Introductory text of the question: Sweden is the only country in the EU that has legislation that means that emergency research that specifically includes drugs cannot be carried out. This is due to a wording in the Medicines Act that differs from how it is formulated in the Ethical Review Act.*

How would you view the fact that we in Sweden have legislation that means that we cannot carry out certain medical emergency research?

- Very positive
- Rather positive
- Not positive nor negative
- Rather negative
- Very negative
- Don’t know

### Original questionnaire in Swedish

**FRÅGEFORMULÄR**

**Om forskning på intensivvårdspatienter**

Inledande text som visats för respondenterna: Vänligen läs noga igenom följande bakgrundsinformation och gå sedan vidare till första frågan: Medicinsk forskning utförs för att få bättre behandlingar och minska risken för biverkningar. Det kan vara läkemedel som skall studeras eller en ny behandlingsmetod. Alla projekt måste granskas av etikprövningsmyndighet som bedömer risker och nytta. Om det är läkemedel som utvärderas granskas projekten också av Läkemedelsverket.

Grundregeln är att patienter som ingår i ett forskningsprojekt så långt som möjligt informeras om forskningen och ges möjlighet att samtycka till, eller motsätta sig, deltagande. I vissa fall – exempelvis när en patient är medvetslös eller mycket kritiskt sjuk – finns det ingen möjlighet att informera eller tillfråga patienten. Även i dessa situationer krävs det forskning för att utveckla och förbättra vården.

I Etikprövningslagen finns det därför ett undantag från information och samtycke där man i dessa situationer kan samråda med en anhörig för att försöka bilda sig en uppfattning om patientens inställning. Läkemedelslagen är däremot skriven så att det inte alls går att göra forskning, om patienten är akut sjuk eller medvetslös. Behandlingar och läkemedel som vi använder för akut sjuka i Sverige, bygger därför till stor del på forskning och kunskap från andra länder.

I en del av de följande frågorna ber vi dig tänka dig in i en situation där du har blivit så allvarligt sjuk att du själv inte kan förmedla din vilja (till exempel vid en stroke, hjärtinfarkt eller svår olycka), och i en del att du istället är anhörig till en patient som är allvarligt sjuk på motsvarande sätt.

*Inledande text till första frågan: Föreställ dig att du skulle få covid-19 (Corona) och behöva vård på sjukhus. Ditt tillstånd blir kraftigt försämrat när du kommer till sjukhuset. Läkarna söver dig och kopplar dig till en respirator. Det finns ett nytt läkemedel som utvärderas i en läkemedelsstudie med en förhoppning om att det ska kunna lindra sjukdomen och kanske minska risken för död. Ingen vet om läkemedlet har effekt på just dig och det finns en del biverkningar som med de flesta läkemedel. Studien har fått ett etiskt godkännande eftersom man bedömt att nyttan överväger riskerna. När ditt tillstånd förbättrats kommer du att bli informerad och få möjlighet att uttrycka din vilja om att delta i projektet framöver.*

**FRÅGA 1**: I ett läge där du inte är vid medvetande, hur ställer du dig till att delta i en medicinsk forskningsstudie, trots att du inte kan bli informerad om den eller ge ditt samtycke?

- Skulle absolut vilja delta
- Skulle troligen vilja delta
- Skulle troligen inte vilja delta
- Skulle absolut inte vilja delta
- Vet ej

**FRÅGA 2**: I ett läge där du inte är vid medvetande, hur ställer du dig till att en närstående/anhörig försöker förmedla din vilja angående om du skulle vilja delta i en medicinsk forskningsstudie eller inte? Din närstående beslutar inte, men forskarna önskar höra vad din närstående tror att du skulle vilja. I lagtexten beskrivs detta som ett samråd.

- Mycket positiv
- Ganska positiv
- Varken positiv eller negativ
- Ganska negativ
- Mycket negativ
- Vet ej

**FRÅGA 3**: I ett läge där du inte är vid medvetande, hur ställer du dig till att en professionell person, exempelvis en jurist eller läkare, godkänner eller avslår att du deltar i medicinsk forskning?

- Mycket positiv
- Ganska positiv
- Varken positiv eller negativ
- Ganska negativ
- Mycket negativ
- Vet ej

*Inledande text till denna fråga: I många länder i EU, undantaget Sverige, kan anhöriga/närstående besluta om en patient, som inte själv kan förmedla sin vilja, kan delta i en studie eller ej.*

**FRÅGA 4**: Hur ställer du dig till att en närstående godkänner om du kan delta i en medicinsk forskningsstudie eller inte, om du inte själv kan förmedla din vilja? (Inte bara samråd, utan din närstående ger sitt samtycke till att du deltar eller svarar nej till att du deltar).

- Mycket positiv
- Ganska positiv
- Varken positiv eller negativ
- Ganska negativ
- Mycket negativ
- Vet ej

*Inledande text till denna fråga: Föreställ dig att du skulle få ett hjärtstopp, återupplivas men förbli medvetslös under ett dygn innan du återfår medvetandet. I ambulansen har man en ny utrustning för att förbättra möjligheten till att göra hjärt-lungräddning. Denna utrustning skall testas i en stor studie för att se om den är säker och effektiv. Tidigare har mindre studier på människa och djurstudier visat positiva resultat, men det är inte helt bevisat att apparaten gör nytta, kanske har den ingen effekt alls eller så ger den mer biverkningar än nytta. Det är därför forskningen skall göras. För att kunna utvärdera effekten av apparaten måste ambulanspersonalen omedelbart bestämma om du skall vara med i studien. Det finns ingen tid att vänta eller fråga någon anhörig. Etikprövningsmyndigheten har givit sitt godkännande till studien.*

**FRÅGA 5**: Hur ställer du dig till att delta i en sådan studie, trots att du inte kan bli informerad om den eller ge ditt samtycke eftersom du är medvetslös och det dessutom inte finns tid att samråda med en närstående?

- Skulle absolut vilja delta
- Skulle troligen vilja delta
- Skulle troligen inte vilja delta
- Skulle absolut inte vilja delta
- Vet ej

*Inledande text till denna fråga: Du blev inkluderad i forskningsstudien i ambulansen och du har nu lagts in på sjukhus. Forskningen har alltså redan påbörjats eftersom den inte kunde vänta.*

**FRÅGA 6**: Hur ställer du dig till att en närstående i efterhand försöker förmedla din vilja om du skulle vilja fortsätta att delta i den medicinska forskningsstudien eller inte? Din närstående beslutar inte, men forskarna önskar höra vad din närstående tror att du skulle vilja (samråd i efterhand).

- Mycket positiv
- Ganska positiv
- Varken positiv eller negativ
- Ganska negativ
- Mycket negativ
- Vet ej

**FRÅGA 7**: Vem, om någon, anser du bäst representerar din vilja om att delta i medicinsk forskning i en situation när du själv inte kan ge uttryck för dina åsikter? Ange maximalt tre svar

- Make/maka/sambo/partner
- Barn
- Föräldrar
- Syskon
- Läkare jag träffat tidigare, t ex husläkaren
- Vän
- Oberoende läkare
- Annan släkting än föräldrar/barn/syskon
- Juridiskt ombud
- Annan person än ovanstående
- Det finns ingen som kan meddela min vilja, men Jag kan tänka mig att delta i forskning
- Det finns ingen som kan meddela min vilja, och jag kan inte tänka mig att delta i forskning
- Vet ej

**FRÅGA 8**: I ett läge där en närstående inte är vid medvetande, hur ställer du dig till att du som närstående/anhörig till en patient försöker förmedla din närståendes vilja att delta i medicinsk forskning? Du beslutar inte för din närstående, men forskarna önskar höra vad du tror att din närstående/anhörige skulle vilja (samråd).

- Mycket positiv
- Ganska positiv
- Varken positiv eller negativ
- Ganska negativ
- Mycket negativ
- Vet ej

**FRÅGA 9**: I ett läge där en närstående inte är vid medvetande, hur ställer du dig till att du som närstående till en patient godkänner om din närstående/anhörige kan delta i medicinsk forskning eller inte? (Inte bara samråd, utan du ger ditt samtycke till att din närstående/anhörige deltar eller svarar nej till att din närstående/anhörige deltar).

- Mycket positiv
- Ganska positiv
- Varken positiv eller negativ
- Ganska negativ
- Mycket negativ
- Vet ej

*Inledande text till denna fråga: Idag kan man upprätta en så kallad framtidsfullmakt som ger anhöriga möjlighet att sköta personliga eller ekonomiska frågor om den ena parten blir oförmögen att själv fatta beslut, som exempelvis vid akut sjukdom. Den gäller dock inte för medicinska frågor.*

**FRÅGA 10:** Hur ställer du dig till möjligheten att upprätta en fullmakt där du i förväg kan dokumentera att en närstående/anhörig får besluta i medicinska forskningsrelaterade frågor om du själv inte kan besluta?

- Mycket positiv
- Ganska positiv
- Varken positiv eller negativ
- Ganska negativ
- Mycket negativ
- Vet ej

**FRÅGA 11**: Viss medicinsk forskning kan på grund av nuvarande lagstiftning inte utföras på akut sjuka patienter i Sverige. Hur ställer du dig till att vi använder resultat som framkommit i andra länder där lagstiftningen tillåter sådan forskning?

- Mycket positiv
- Ganska positiv
- Varken positiv eller negativ
- Ganska negativ
- Mycket negativ
- Vet ej

*Inledande text till denna fråga: Sverige är det enda land i EU som har en lagstiftning, som innebär att akutforskning som just omfattar läkemedel inte kan genomföras. Detta beror på en formulering i Läkemedelslagen som skiljer sig från hur den är formulerad i Etikprövningslagen.*

**FRÅGA 12**: Hur ser du på att vi i Sverige har en lagstiftning som innebär att vi inte kan genomföra viss medicinsk akutforskning?

- Mycket positiv
- Ganska positiv
- Varken positiv eller negativ
- Ganska negativ
- Mycket negativ
- Vet ej
